# Scoping Review About *Salmonella* spp. in Colombian Pig Farms from 2009 to Mid-2024

**DOI:** 10.3390/ani14233542

**Published:** 2024-12-08

**Authors:** Adriana Pulido-Villamarín, Iliana Chamorro-Tobar, Ana K. Carrascal-Camacho, Fernando Sampedro, Marcela Rodríguez-Moreno, Fernando Rojas-Bermúdez, Mónica Pérez-Vargas, Ivonne Hernández-Toro, Alejandra Camacho-Carrillo, Raúl A. Poutou-Piñales

**Affiliations:** 1 Unidad de Investigaciones Agropecuarias (UNIDIA), Departamento de Microbiología, Facultad de Ciencias, Pontificia Universidad Javeriana, Bogotá 110231, Colombia; 2 Grupo de Biotecnología Ambiental e Industrial (GBAI), Departamento de Microbiología, Facultad de Ciencias, Pontificia Universidad Javeriana, Bogotá 110231, Colombiarpoutou@javeriana.edu.co (R.A.P.-P.); 3 Asociación Porkcolombia—Fondo Nacional de la Porcicultura, Centro de Investigación y Transferencia de Tecnología del Sector Porcícola (CENIPORCINO), Bogotá 110231, Colombia; 4 School of Public Health, Environmental Health Sciences, University of Minnesota, Minneapolis, MN 55455, USA; 5 Área de Bacteriología, Laboratorio Nacional de Diagnóstico Veterinario (LNDV), Instituto Colombiano Agropecuario—ICA, Bogotá 110221, Colombia; 6 Programa de Microbiología Industrial, Facultad de Ciencias, Pontificia Universidad Javeriana, Bogotá 110231, Colombia

**Keywords:** Colombia, pig farms, pig production, primary production chain, prevalence, risk factors, *Salmonella* spp., seroprevalence

## Abstract

In Colombia, research on Salmonella concerning animal health, veterinary diagnostics, and epidemiology within the primary production chain is limited. This study aimed to analyze the published data about Salmonella in the Colombian primary pig production chain from 2009 to mid-2024. This involved an exploratory literature review using systematic search strategies, including articles, graduate studies, conference presentations, and technical reports from the selected period. Of the 35 studies reviewed, 30 met the inclusion criteria, with eleven being from the grey literature. The pooled prevalence of *Salmonella* spp. on Colombian farms was 8.9%, while the seroprevalence ranged from 27 to 40%. Risk factors associated with the presence of this bacterium on farms included aspects such as water sources, pest control, the farm type, and management practices. Few scientific publications address the presence of this pathogen in primary pig production in Colombia, underscoring the need to raise awareness within the academic and production communities about the importance of conducting and reporting new studies and cases.

## Simple Summary

The global increase in pig production has been economically significant. However, a critical issue is the pathogens affecting the production chain, particularly *Salmonella* spp. This microorganism compromises animal health and growth, resulting in economic losses and posing a public health risk due to potential transmission to humans via contaminated pork product consumption. The lack of epidemiological data about *Salmonella* spp. in Colombian pig farms has become a challenge to effective risk mitigation measures, highlighting an urgent need to improve surveillance and disease control within the production chain.

## 1. Introduction

The worldwide pork production and trade have increased recently, especially in the European Union, Russia, and the United States. Over the last two decades, the global pork production has increased by 111%, with technological innovation being a fundamental factor [1]. Colombian pork production represents 1.4% of the agricultural gross domestic product (GDP) and 4.8% of the livestock sector’s GDP, generating approximately USD 900 million/year [2]. Its production has continued to grow: in 2015, there were 285,280 tons of pork, and, by 2019, there were nearly four million slaughtered pigs, amounting to 406,085 tons of meat [3]. In 2019, the pork production in Colombia amounted to approximately 6,473,525 animals, concentrated mainly in the departments of Antioquia (29.68%), Valle del Cauca (14.36%), Cundinamarca (8.94%), Meta (6.03%), Córdoba (6.01%), Magdalena (4.21%), Sucre (3.33%), Boyacá (3.12%), Atlántico (2.74%), and Bolívar (2.27%) [4].

Pork production is susceptible to various pathogens, including viruses, parasites, and bacteria, which cause serious health issues and economic losses by slowing growth rates, reducing animals’ weight, decreasing the meat quality, and causing some cases of animal mortality. Additionally, the diagnosis and treatment of diseases incur additional expenses that can be large [5]. Pathogens also pose public health risks, particularly foodborne zoonotic pathogens, which can increase human morbidity and mortality. *Salmonella* spp. is one of the primary pathogens that is responsible for around 94 million gastroenteritis cases and 155,000 deaths yearly worldwide [6].

Pigs are a significant reservoir of *Salmonella* spp., being a source of infection for humans when they consume contaminated pork products or come into contact with the animals [7]. This transmission leads to salmonellosis, presenting symptoms such as diarrhea, fever, and abdominal cramps, with severe cases causing arthritis, pneumonia, meningitis, and septicemia [8]. The European Food Safety Authority’s (EFSA) studies reported that 8.9% of salmonellosis cases were due to consuming contaminated pork or pork by-products [9]. In the United States, the reported values were between 3 and 10% in 2015–2017 [10,11], in which there were 281 outbreaks related to the intake of contaminated pork and its by-products [12]. Similarly, in Colombia, there were 867 outbreaks of foodborne diseases in 2017, with *Salmonella* spp. as the etiological agents in 15 cases [13]. The INS clarified that the FBD incidence has a direct relationship with food quality, especially when the contamination occurs with the same organic matter of the animal, as many human pathogens are considered normal microbiota in protein-source animals. The INS reported that, of the 881 outbreaks detected in 2018, beef and pork meat were involved in 6% of them [14].

Salmonella is a genus within the Enterobacteriaceae family, consisting of Gram-negative bacilli that are facultative anaerobes and grow optimally at 37 °C. It has been found in reptiles, birds, and rodents, among other wildlife specimens, which behave as natural reservoirs of the pathogen and can sporadically enter farms [15]; on the other hand, the bacterium can persist in the environment for extended periods, contaminating water, feed, and surfaces [16,17,18]. Once Salmonella infects pigs, it causes asymptomatic infections, allowing subclinical carrier animals to serve as a contamination source for the healthy populations of farms [19] and processing plants [20]. Worldwide, the average on-farm prevalence has oscillated between 17 and 59% [21,22]. However, in Latin America, and specifically in Colombia, epidemiological data regarding the presence of this microorganism in swine production farms are scarce, which hinders the implementation of plans to reduce the zoonotic risk in the primary chain.

Given the economic and public health significance of pig production, the present research team aimed to analyze publications on *Salmonella* spp. in the pig production chain of Colombia between 2009 and mid-2024. This study followed the PICoR scheme (Patient/Problem, Intervention, Comparison/Alternative vs. Standard, Reports), addressing the following question: what has been published about *Salmonella* spp., within the primary chain of Colombian pig production, from 2009 to mid-2024? However, the answer may not fully capture the reality of the situation regarding *Salmonella* spp. in Colombian pig farms due to underdiagnosis and a lack of official reporting. The team hopes this exploratory review will promote awareness and potentially encourage more rigorous surveillance and reporting practices within Colombia and similar contexts.

## 2. Materials and Methods

### 2.1. Protocol

This analysis was conducted according to the methods outlined by previously published strategies [23,24].

### 2.2. Search Strategy

A literature review was conducted by searching databases such as ScienceDirect, SciELO, EBSCOhost, Redalyc, Google Scholar, and all grey literature derived from associations and governmental institutions, corresponding to the period between 2009 and mid-2024. The MeSH search terms were Salmonella “AND” Colombia “AND” farm “AND” pig/swine/pork and Salmonellosis “AND” Colombia “AND” pig/swine/pork “AND” farm, in Spanish, English, and Portuguese.

### 2.3. Inclusion and Eligibility Criteria

Based on the acronym PICoR (Table 1), the inclusion and eligibility criteria concerned all publication types/documents between 2009 and mid-2024 related to *Salmonella* spp. in the Colombian primary pig production chain. There was no restriction on the age of the animals, the place or region, or the type of study reported.

### 2.4. Data Extraction

The Microsoft Excel software allowed for the collection of information such as the author, year, production stage, age, place or region, type and number of samples, technique, methodology, seroprevalence, prevalence, serotypes, and other results.

The prevalence analysis reported in all papers considered in this study referred to the traditional microbiological method of the pre-enrichment and handling of selective and differential media, with confirmation by molecular methods (MDS 3M^®^ and PCR) in three of the reports. The methodology used for seroprevalence determination was ELISA.

**Figure 1:**
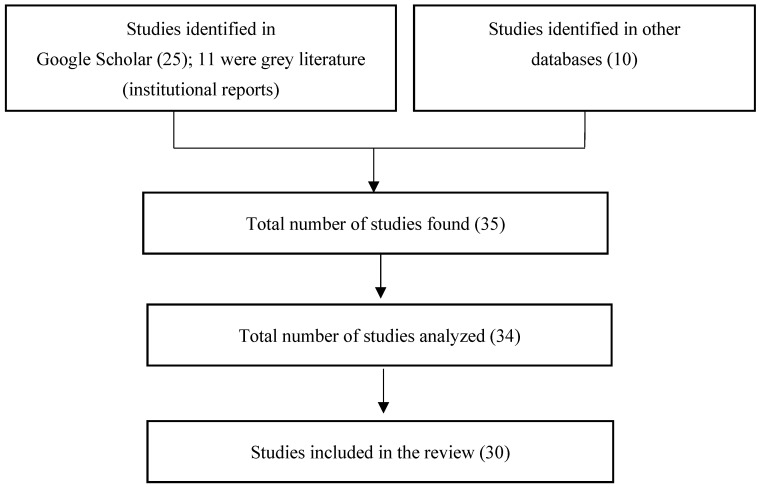
PRISMA flowchart of the procedure for the selection of the studies analyzed.

Once the data were tabulated, we carefully reviewed the abstracts, procedures, and results from all documents that met the inclusion criteria. Two researchers independently reviewed the abstracts and selected those relevant for this review. Figure 1 illustrates the procedure performed to select and discard bibliographic sources. No duplicated documents were included.

### 2.5. Analysis

Fifteen selected articles (50%) containing prevalence and seroprevalence data served for analysis. The meta-analysis methodology previously described, using the MetaXL V5.3 software (Epigear International, Sunrise Beach, Australia), was used to calculate the combined prevalence of all of the studies from the literature search in Instituto Colombiano Agropecuario [20]. To this end, we used three models from the meta-analysis methodology, i.e., fixed effects, random effects, and inverse variance heterogeneity models, to estimate the data heterogeneity and select the most appropriate model to estimate the combined prevalence and seroprevalence of *Salmonella* spp. in Colombian pig farms.

## 3. Results

Related to the searches performed in the scientific databases, only Scielo and Google Scholar yielded results for the keywords used. Additionally, we included information from other sources, such as technical reports, undergraduate theses, etc., corresponding to 11 grey literature documents.

Based on the information analyzed, we found that clinical, serological, microbiological, and molecular studies on the detection of *Salmonella* spp. have been conducted throughout the primary production cycle in different departments in the national territory, which we will discuss later in the corresponding section.

### 3.1. Pig Salmonellosis

**Figure 2:**
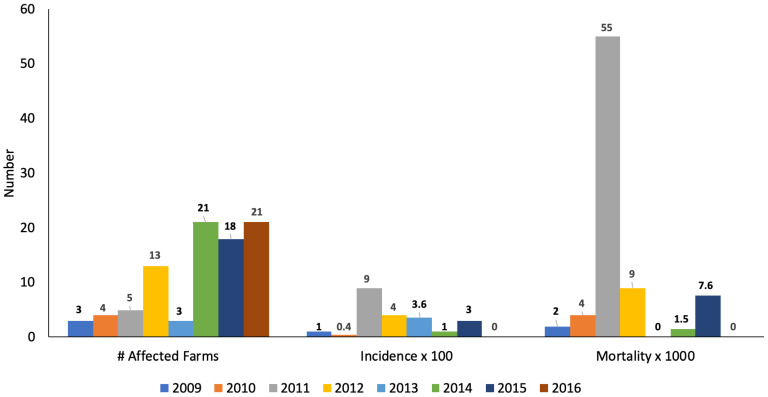
Swine salmonellosis is an infectious pathological condition reported in Colombia, according to the parameters of several affected farms and its incidence and mortality.

In Colombia, the ICA (animal health regulator) has reported salmonellosis as an infectious pathological condition. Figure 2 shows the data on the affected farms and consolidated epidemiological data from 2009 to 2016 [25,26,27,28,29,30,31,32].

On the other hand, one of the clinical reports on the disease in Antioquia showed weak animals with evidence of anoxia (“purple ears and extremities”) in the rearing stage and animals with “liquid” diarrhea, with the subsequent mortality of three piglets within 24 h of this clinical evidence; the symptomatology increased with time, necessitating the administration of trimethoprim sulfa (sulfatrim). The mentioned study reported a morbidity rate of 90% and a mortality rate of 10%, with the diagnosis of salmonellosis confirmed by the isolation of the pathogen from the spleen, cecum, mesenteric lymph nodes, intestinal abscesses, and feces [33]. In the same department, animals with clinical signs of *Salmonella* spp. were treated with trimethoprim sulfa and enrofloxacin; however, there were repeated cases, suggesting resistance to these antibiotics, especially to trimethoprim sulfa [34]. In the Atlantic region of the country, the microorganism’s isolation was positive in 33% of the farms, and the authors found weaned piglets with symptoms compatible with salmonellosis (diarrhoea). All isolates were susceptible to the antibiotics tested (ampicillin, cefotaxime, ceftazidime, ceftriaxone, ciprofloxacin, and trimethoprim/sulfamethoxazole) [35].

### 3.2. Epidemiological Parameters

**Figure 3:**
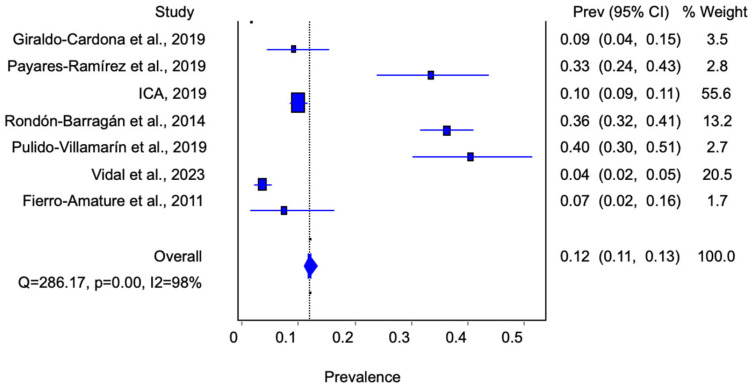
Estimation of *Salmonella* spp. prevalence (P) in Colombian pig farms (the percentage weight is in the order 3.5; 2.8; 55.6; 13.2; 2.7, and 1.7 and an overall value of 100.0) [16,17,35,36,37,38,39].

**Figure 4:**
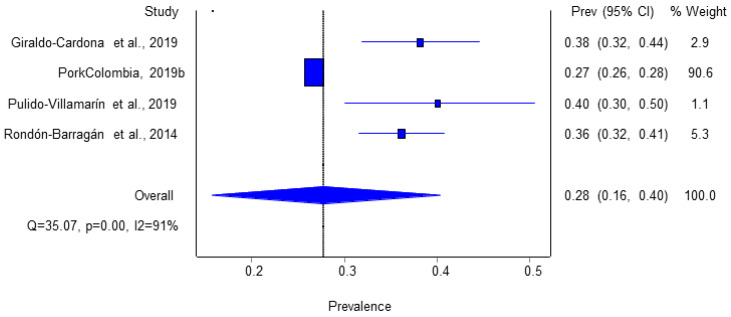
Estimation of *Salmonella* spp. seroprevalence (SP) in Colombian pig farms [3,16,37,38] 2014.

**Figure 5:**
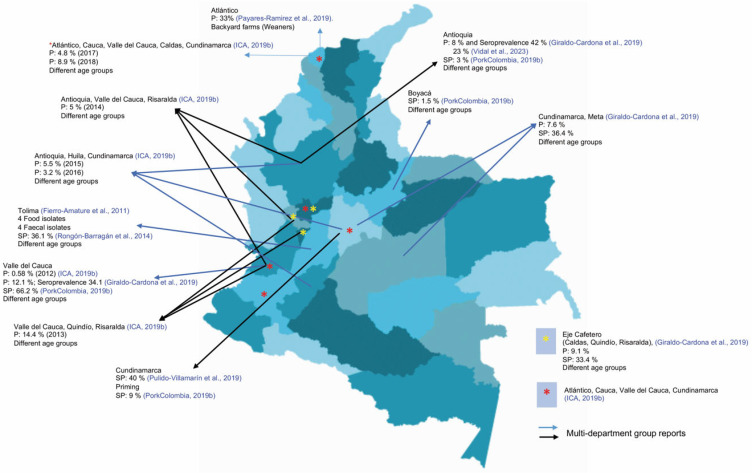
Map of prevalence (P) and seroprevalence (SP) against *Salmonella* spp. as reported in primary production throughout the national territory from 2009 to mid-2024 [3,16,17,35,36,37,38,39].

Prevalences (P) and seroprevalences (SP) determined by microbiological, serological, and molecular tests have been reported throughout the national territory (Table 2, Table 3 and Table 4). The P data ranged from 0.58 to 28% in several departments. These included unpublished data from the Colombian national casuistry and a report for internal circulation only [36]. Moreover, the SP averaged 29% [16,37,38] (internal circulation report (Porkcolombia)) (Figure 3, Figure 4 and Figure 5).

**Table 2:** *Salmonella* spp. prevalence (P) reported in Colombian pig farms.

Reference	Year of Origin	Geographic Location	No. Samples Analyzed	No. Positive Samples	% P
[16] *		Colombia	504	45	8.0
[35]		Valledupar, Atlántico	90	30	33.0
[36]	(Data from 2018 *)	Colombia	385	108	28.0
	(Data from 2018 *)	Colombia	203	18	8.9
	(Data from 2017 *)	Colombia	147	7	4.8
	(Data from 2016)	Cundinamarca, Antioquia	93	3	3.2
	(Data from 2015 *)	Colombia	273	15	5.5
	(Data from 2014 *)	Colombia	238	12	5.0
	(Data from 2013 *)	Colombia	88	10	11.4
	(Data from 2012)	Valle del Cauca	344	2	0.58
[17]		Antioquia	653	149	23

* More than three Colombian Departments analyzed.

**Table 4:** Seroprevalence (SP) of *Salmonella* spp. reported in Colombian pig farms.

Reference	Geographic Location	No. Samples Analyzed	% SP
[16]	Colombia	231	38.1
(Internal circulation report (Porkcolombia))	Colombia	7140	26.7
[38]	Cundinamarca	89	40.0
[37]	Tolima	420	36.1

The above depicts each study’s estimated prevalence, ranging from 1 to 33%. In turn, the work conducted by the ICA had the highest relative weight concerning the combined prevalence calculation, with 55.6%. However, the sensitivity analysis showed that no study significantly affected the estimation of the combined prevalence [16] (Figure 3).

Table 3 shows the combined probability estimated with the three models. As can be seen, the value of Cochran’s Q test for heterogeneity (Q = 286.17) and the inverse variance index (I2 = 98) was greater than 75%, indicating high heterogeneity among the studies. According to Barendregt et al. (2013), the inverse variance heterogeneity model should be used to analyze high-heterogeneity data. In this study, the model yielded a pooled prevalence value of 12.0% (confidence interval (CI), 10.9–13.1) [40].

The inverse variance heterogeneity model reported a pooled seroprevalence value of 27.6% (CI, 15.8–40.3). Figure 4 shows the estimated seroprevalence of each study, which ranged between 27 and 40% (unpublished data from the Colombian national casuistry). The report for internal circulation only [36] showed the highest relative weight concerning the calculation of the combined seroprevalence (90.6%) due to the number of samples analyzed. It is crucial to remark that the studies that reported P and SP data were conducted using validated detection techniques for both the bacterium and the antibodies against it.

The report from the Colombian Association of Pig Farmers—National Fund for Pig Farming (Asociación Colombiana de Porcicultores—Porkcolombia—Fondo Nacional de la Porcicultura (FNP)) indicates that, for the period between 2014 and 2018, when employing voluntary routine diagnosis in Colombian farms, the national average seroprevalence was 26.7%. The results by department evidenced that the highest value was found for Valle del Cauca, with 66.2%, followed by Caldas at 14.7%, Cundinamarca at 9%, Antioquia and Cauca at 3%, Quindío at 2.7%, and Boyacá at 1.5%; 2018 was the year with the highest seroprevalence (53.9%). The high seroprevalence was due to the producers’ awareness of animal health and public health problems through research conferences. In 2018, more samples were sent to diagnostic laboratories, with 3406 samples received (samples)—a value that increased over time (2014: 30 samples, 2015: 726 samples, 2016: 893 samples, 2017: 2055 samples) [36].

Concerning seropositivity according to the age group, 40.9% of the positivity occurred in the growing-finishing stages, 24.8% in the nursery, 20.1% in sows, and 6.1% in replacement gilts [37].

At the national level, there are diverse production systems and different types of farms. According to the parameters established by Porkcolombia, following the sanitary design submitted to the World Organization for Animal Health (WOAH) for notifiable diseases, and based on the 2018 census, the findings for the farms by type of exploitation definition were as follows: (i) backyard—there were 23,539 holdings, ≤3 female breeding piglets (FPB) or ≤15 fat; (ii) commercial family—with 92,572 holdings, >3 and <10 HC and or >15 and <100 fat; (iii) industrial commercial—with 7659, owning between ≥10 and <100 HC and/or ≥100 and <600 fat; and (iv) commercial technical (Colombian denomination) —with 9122, those with ≥100 HC and/or ≥600 fat.

The farms by the type of production system and production site are named as follows: full-cycle farms (FC); multi-site farms (MSF); farms with mothers for piglet rearing (piglet); and farms where animals are raised and fattened (fattening).

On the other hand, according to the flow of animals on the farm, there is the continuous flow (CF) and the all-in/all-out (AIAO) production system. There are two production lines: the first is the production, which refers to the nursery, growing, and finishing stages, and the reproduction line refers to sows, piglets, barrows, and replacement gilts. According to these production parameters, the percentages of SP obtained through the routine diagnosis carried out by Porkcolombia were different (Table 5).

### 3.3. Serovars and Antimicrobial Resistance (AMR)

The reports found in Colombia indicate the following the predominant serovars: *S*. Typhimurium, *S*. Brancaster, *S*. Derby, *S*. Typhimurium variant Copenhagen, *S*. Heidelberg, *S*. Group B, *S*. Group E1, *S*. Group D2, and *S*. Group F. [36,39], with the presence of *S*. Typhimurium as a monophasic variant in the last few years [17].

The isolates obtained from Colombian farms show different antimicrobial susceptibility profiles, with evidence of sensitivity to amikacin (100%) and resistance close to 95% for penicillin, lincomycin, and tetracycline [16,39], among other antibiotics. On the other hand, in recent years, multi-resistance rates (resistance to three or more antibiotic classes) have been reported between 44 and 70.3% [17,37,41].

### 3.4. Risk Factors

In Colombia, there are few studies carried out on issues other than sanitary and epidemiological aspects. In 2012, a study reported the risk factors associated with the presence of the bacterium on farms in Tolima; in this study, some parameters were established, such as the control of rodents, insects, and birds. New animals and self-replacements were significantly associated with the presence of the pathogen on farms [42]; however, no further specific mention was made of the characteristics of the variables analyzed. Furthermore, a consolidated report for the four regions of Colombia where pig production is predominant (Antioquia, Valle del Cauca, Eje Cafetero, Cundinamarca-Meta) concluded that the factors associated with the presence of the bacterium on farms are related to the source of water supplied to the pigs [43]; rough flooring in the animal pens; the feed supplied in hoppers; and workers’ boots [16].

On the other hand, analyzing the data related to the risk factors, Porkcolombia indicates the possibility of increasing the presence of antibodies against *Salmonella* spp. with a statistically significant association. These factors include (a) the type of farm, where those with a complete cycle exhibited a 27.9 times higher likelihood of presenting antibodies against *Salmonella* spp. (odds ratio (OR) 27.9; 95% CI 5.79–315.6) concerning fattening farms; (b) the flow of the farm, as those farms with an “all in/all out” system showed a 24 times higher likelihood (OR 24.9; 95% CI 5.22–272. 39) of seropositivity concerning continuous-flow farms; and (c) the type of farm, where “commercial technical” farms (farms with ≥100 breeding females and or ≥600 heads) had an OR of 1.92 (95% CI 1.18–3.13), which, although relatively low, is a relevant factor when establishing prevention and control measures [36].

### 3.5. Water and Organic Waste Management

Some studies have reported pathogen studies related to the environmental management of waste from pig production and water. The first one was related to the management and quality of pig manure in Caldas, in which the fermentation process favored the reduction of the Salmonella load, ensuring the safety of silage supplemented with pig manure [44]. In Antioquia, the process of composting pig manure and pig mortality using a bioinoculant accelerator favors the production of compost free of bacterial pathogens (including Salmonella) and parasites in a shorter time (30 days) compared to the traditional method (45 days) [45].

Regarding water management, a study in Cundinamarca showed the effectiveness of photolysis and photolysis with hydrogen peroxide in pig farm water for the inactivation of the microorganism [43]. In Antioquia, the additives (Selko 0.15–0.3 mg/L) used in the water showed promising results in reducing the symptoms [34]; similarly, in 2023, it was found that the maintenance and cleaning of water pipes and the administration of organic acids in pigs’ drinking water favored the productivity of the animals and delayed their exposure to the bacterium [46].

## 4. Discussion

Clinically, salmonellosis in pigs generates gastrointestinal, systemic, and occasionally subclinical symptoms, making the detection of carrier animals difficult and facilitating the pathogen’s spread on farms, because Salmonella persists in the animal’s spleen and mesenteric lymph nodes [19,47]. This situation has been observed in several departments in Colombia, where subclinical carrier animals are likely to maintain the pathogen within the farm population [16,36,37].

The disease may appear at any stage of the production cycle, although its severity is generally more pronounced in younger animals [48], such as suckling piglets, where diagnosis is limited to obtaining rectal swabs, thus complicating accurate diagnosis and allowing these animals to become risk factors for the maintenance of bacteria on farms [49].

The information reported confirms the importance of piglets in the maintenance of the microorganism within the farm, facilitating its rotation in nursing mothers [26,36]; however, in the data reported for Colombia, the pre-feeding and rearing–weaning stages presented the highest seropositivity values [44].

On the other hand, although the treatment of salmonellosis by antibiotics is recognized worldwide, as reported in Colombian studies, an Argentinian work evaluated extracts obtained from lactic acid bacteria (LAB) isolated from the colostrum of mothers as a treatment alternative, demonstrating inhibitory effects “In Vitro” [39]. On the other hand, with the preventive management of antibiotics and the use of pH-modifying additives, the symptomatology decreased, a situation that was achieved by the addition of organic acids such as propionic acid (0.8%), formic acid (0.2%), or lactic acid (0.4%) to feed or drinking water [42,50]. This is a practice that has yielded good results as a prevention and control strategy against salmonellosis worldwide.

While Colombian studies have not yet explored the efficacy of animal vaccination against Salmonella, international findings suggest that the vaccine’s effectiveness varies depending on the products and protocols used. Live vaccines using *S.* Typhimurium and *S.* Choleraesuis serovars are prevalent, while inactivated vaccines are less common; however, both have been considered effective in reducing infection rates and minimizing the contamination of pork products intended for human consumption [42,44].

Epidemiological data related to P and SP were calculated and analyzed based on the laboratory tests performed for their detection, either because the bacterium was recovered/isolated by microbiological methods or if using serological methods, the antibody response was positive. Such positivity was detected in pigs of different age groups; however, the highest proportion of positivity was reported in adult animals, as corroborated by some studies [51,52].

In Colombia, the combined prevalence was 21.3%, and the seroprevalence was 27.6%. Worldwide, P and SP are usually high [19]; however, reports for farms in different countries have been variable, e.g., Germany reported 7.9% (SP) [53], Romania 35.8% (SP) [53], 36% (P) for Canada [51], and 41.5% (P) in Argentina [54]. These data suggest that the management conditions and prevention and control programs are different in each country.

Although more than 2000 serovars are recognized within the *Salmonella enterica* species [6], many of them tend to be host-specific. Thus, *S. enterica* serovar Cholerasuis is usually associated with disease in pigs; however, *S. enterica* serovar Typhimurium cannot be overlooked as it can also be clinically relevant [19], and, being a non-host-specific serovar, it represents a major public health concern due to its zoonotic transmission.

Internationally, *S*. Typhimurium and *S*. Derby are the most frequently reported serovars in studies related to animals and farms [21,53]; compared to this, the data found for Colombia do not differ from the serovars that have predominated, such as *S*. Typhimurium, *S*. Brancaster, *S*. Derby, *S*. Typhimurium variant Copenhagen, and *S*. Heidelberg, as well as a few others [38,51]. This information also coincides with the serovars found along the primary chain in Canadian farms (*S*. Derby, *S*. Typhimurium var. Copenhagen, *S*. Putten, *S*. Infantis, and *S*. Mbandaka) [52,55]; for Latin America, specifically in Argentina, *S*. Anatum and *S*. Typhimurium have predominated, with the detection of the monophasic strain S. 4, 5, 12:i: [54]. Although, for Colombia, specific data on the serotypes found by age group and the presence of the monophasic serovar of *S*. Typhimurium (*S*. 4, 5, 12:i:) have been reported, they highlight the necessity to expand field studies. In Spanish farms, its presence has been detected together with *S*. Rissen, *S*. Derby, and *S*. Bovismorbificans in weaned piglets and their mothers, leading to the establishment of the bacterium in these farms [26]. Furthermore, the detection of the mentioned serovar indicates a high degree of antimicrobial resistance [53,56], either by intrinsic genetic factors or acquired through plasmids; thus, in Colombia, it is necessary to be alert at the time of its appearance to take control and prevention measures.

The resistance to penicillin, lincomycin, and tetracycline [16,17,39,41] could be similar to that found in Brazil [51], Argentina [52], Portugal [53], and, in general, the European Union [54], suggesting that the indiscriminate use of antibiotics throughout the primary production chain could favor this species, enabling it to become a reservoir of multi-resistant strains [55].

According to Davies [11,57], one- to three-site AIAO production systems represent higher protection and better control for pig health than FC farms and can prevent cross-contamination between production cycles by facilitating thorough cleaning and disinfection, reducing the potential for exposure to Salmonella infection [11,57]. In Colombia, AIAO systems and practices are frequent; however, in routine diagnosis, a statistically significant association (*p* = 0.05) was found between AIAO/FC and the presence of the bacterium. In other countries, some of the risk factors associated with *Salmonella* spp. on farms include the inadequate handling of operators’ boots, the contamination of water drains [55], and nose-to-nose contact between animals in the same pen [51], as well as feed prepared with contaminated raw materials and possibly “pelleted” food [43,52]. Therefore, the risk factors described in national and international scientific publications agree with those analyzed by Porkcolombia [38].

## 5. Conclusions

In summary, the estimated combined prevalence for *Salmonella* spp. in Colombian pig farms is 21.3%, while the seroprevalence is 27.6%, with the production systems and animal flow significantly influencing the pathogens’ presence. Data on *Salmonella* spp. in Colombia’s primary pork production chain remain scarce, with most reports categorized as grey literature.

Although *Salmonella* spp. are leading zoonotic pathogen responsible for foodborne outbreaks traced to animal products, including pork, there is a lack of official reporting for these pathogens in Colombia’s primary production chain. It is essential to raise awareness within the government and academic circles regarding the importance of reporting epidemiological data on pathogens that affect animal production and pose risks to human health.

## Figures and Tables

**Table 1:** Eligibility criteria definitions according to PICoR approach.

Acronym	Criteria
P (Population/Patient/Problem)	Studies and reports obtained from the pig production chain at the primary scale in Colombia.
I (Intervention)	Studies and reports related to *Salmonella* spp. and salmonellosis.
Co (Comparison, Outcomes, Alternative vs. Standard)	Studies and reports related to prevalence, seroprevalence, pathology, risk factors, and farm management.
R (Reports)	Studies and reports published in English or Spanish throughout the country during the years 2009–2024.

**Table 3:** Pooled prevalence and confidence intervals for the fixed effects model, random effect model, and inverse variance heterogeneity model.

Model Type	Combined Prevalence	LCI *	HCI *	Range **
Fixed effects	0.120	0.109	0.131	0.022
Random effect	0.172	0.075	0.295	0.22
Inverse variance heterogeneity	0.120	0.029	0.289	0.25

* LCI and HCI are low (2.5%) and high confidence intervals (97.5%). ** The range is the difference between the minimum and maximum values.

**Table 5:** Percentages of seroprevalence (SP) according to production parameters, through routine diagnosis [36].

Production Parameters	% SP
*Type of Operation*	
Full cycle	97.7
Multiple sites	0.0
Breeding	0.9
Fattening	1.4
*On-Farm Flow*	
Continuous flow—FC	100.0
All in/all out—AIAO	0.0
*Property Type*	
Commercially technified	95.7
Commercial industrial	4.3
*Production Line*	
Production	64.0
Reproduction	36.0

## Data Availability

Data are available upon request.
